# Effect of Myocilin E414K Variant on the Pathogenesis of Primary Open-Angle Glaucoma

**DOI:** 10.7759/cureus.83372

**Published:** 2025-05-02

**Authors:** Muhammad Awan, Eric B Johnson

**Affiliations:** 1 Research, Alabama College of Osteopathic Medicine, Dothan, USA; 2 Anatomy and Molecular Medicine, Alabama College of Osteopathic Medicine, Dothan, USA

**Keywords:** genetic mutation, glaucoma, myocilin, primary open-angle glaucoma, variant of uncertain significance

## Abstract

Introduction

Primary open-angle glaucoma is a chronic, multifactorial condition of optic neuropathy. While the exact cause of the disease is not well understood, mutations in the protein myocilin, particularly within the olfactomedin (OLF) domain, have been associated with the development of increased intraocular pressure and open-angle glaucoma. However, many variants of uncertain significance of myocilin remain, with unknown pathogenic roles. In this study, we performed a simulated analysis of a glutamate-to-lysine substitution at position 414 (E414K) in the OLF domain of myocilin to explore the potential role of the variant in the pathogenesis of primary open-angle glaucoma.

Methods

The native and variant myocilin proteins were run through 20-nanosecond molecular dynamics simulations. Structural changes were studied with root-mean-square deviation and dynamic cross-correlation matrix analyses. Predictive programs were utilized to understand the evolutionary patterns of myocilin and estimate the pathogenicity of the E414K substitution.

Results

The root-mean-square deviation analysis revealed no significant structural differences between the native and variant myocilin proteins. While the dynamic cross-correlation matrix heat map suggested alterations in interactions between specific residues in the OLF domain, these changes were not significant enough to disrupt protein function or structure. Evolutionary analysis of myocilin demonstrated that the glutamate residue at position 414 was variable across species, and therefore, mutations at this site are unlikely to be deleterious. PredictSNP (developed by Jiri Damborsky and colleagues at Masaryk University, Brno, Czech Republic) further supported the benign nature of the E414K mutation.

Conclusion

Our study concluded that the E414K substitution is a benign mutation that is unlikely to cause instability of the OLF domain and contribute to the pathogenesis of primary open-angle glaucoma. While limited in scope, our research underscores the importance of further studies into myocilin variants to identify pathogenic mutations. Continued analysis of myocilin variants may advance the understanding and treatment of primary open-angle glaucoma.

## Introduction

Primary open-angle glaucoma (POAG) is a chronic condition of progressive optic neuropathy, characterized by an open anterior chamber angle, optic nerve cupping, and gradual loss of peripheral vision followed by central vision. While the exact cause of POAG is not confirmed, multiple risk factors for glaucoma have been proposed in the literature, including family history, thin corneal thickness, and myopia. Most notably, increased intraocular pressure (IOP) is a well-known risk factor for the development of POAG and is the target of glaucoma therapy [1].

In the normal, healthy eye, the ciliary body produces aqueous humor in the posterior chamber of the eye, which eventually drains into the anterior chamber. Most of the aqueous humor drains through the trabecular meshwork within the iridocorneal angle, with the remainder of the drainage occurring through the uveoscleral pathway [1]. It is theorized that in patients with POAG, there is increased resistance to aqueous humor drainage through the trabecular meshwork despite the iridocorneal angle remaining open. Consequently, IOP increases over time due to the blockage of aqueous humor outflow, resulting in the progressive compression of optic nerve fibers and irreversible vision loss [2].

Mutations of various genes are responsible for a subset of POAG cases, especially in patients with a family history of glaucoma. Myocilin mutations are most associated with open-angle glaucoma, making up around 5% of all hereditary glaucoma cases [3]. A protein product of the MYOC gene on chromosome 1q24.3, myocilin is a multimeric secreted glycoprotein consisting of 504 amino acids (AAs) that is primarily found in the trabecular meshwork of the eyes [4,5]. While its function is not fully certain, it has been hypothesized that myocilin interacts with extracellular proteins (i.e., fibronectin, laminin) to regulate IOP. Namely, the C-terminal olfactomedin (OLF) domain (AA 244-502) of myocilin is involved in the cell adhesion and cytoskeletal organization of trabecular meshwork cells, regulating IOP by influencing aqueous humor outflow [4].

Multiple studies on myocilin variants have supported the conclusion that deleterious mutations in the OLF domain result in the aggregation of insoluble and soluble misfolded proteins within the trabecular meshwork, leading to POAG in millions of hereditary glaucoma cases [3,6]. However, there remain multiple variants of uncertain significance (VUS) of myocilin whose involvement in the pathogenesis of POAG remains unknown [7]. VUS are rare alterations within the human genome with very little information available about their effects or pathogenicity; a variant is classified as a VUS when there is insufficient or conflicting data regarding the mutation's pathogenicity in a certain disease [8].

In the case of myocilin, as of 2021, gnomAD listed 114 variants in the OLF domain with unknown pathogenicity, including a glutamate-to-lysine variant at position 414 [7]. In this brief study, we analyze this glutamate-to-lysine variant of uncertain significance at position 414 (E414K), a substitution within the essential OLF domain of myocilin [4], to determine its effect on the pathogenesis of POAG. Through this brief study, we aim to uncover the pathogenicity of the E414K substitution and promote continued research into other myocilin VUS, in the hope of advancing genetic research into POAG and the definitive treatment of glaucoma.

## Materials and methods

The FASTA sequence of myocilin was accessed on UniProt [9], and the Protein Data Bank (PDB) file containing the structure of the functional OLF domain of myocilin (AA 244-502) was downloaded from the Research Collaboratory for Structural Bioinformatics (RCSB) PDB (PDB identifier: “4WXQ”) [10]. The PDB file was opened on the Yet Another Scientific Artificial Reality Application (YASARA) application [11], and after all water molecules and solvents were removed from the protein, a separate file was then generated where the glutamate at position 414 was replaced with lysine to make the E414K myocilin structure.

The “em_runclean.mcr” macro was run for the PDB files of the native and variant proteins on YASARA, and three replicate PDB files for the native and E414K proteins each were created through energy minimization. 20-nanosecond molecular dynamics simulations of the replicates of myocilin were conducted via the AMBER14 force field [12] to determine how the proteins behave in water and to understand the effects of the AA alteration on the physical properties of myocilin. For the simulation, hydrogen bonding and protonation states were optimized for a pH of 7.4, and sodium chloride (NaCl) was added to reach a concentration of 0.9% [13]. An intramolecular timestep of 1.333 femtoseconds and an intermolecular timestep of 4.0 femtoseconds were applied. The simulation was set to a temperature of 298 K and a pressure of 1 atm. The “md_analyze” and “md_analyzeres” macros were run to obtain the simulation data on YASARA [11].

Following the simulation, the average root-mean-square deviations (RMSD) of the E414K and native replicates across the 20-nanosecond simulations were qualitatively analyzed on Microsoft Excel (Microsoft® Corp., Redmond, WA) to investigate significant structural differences in the OLF domain. The alpha-carbon RMSD averages of the VUS myocilin replicates and native replicates of the last 10 seconds of the simulations (i.e., the point at which protein conformations had stabilized) were also calculated, and the datasets were transposed to produce a two-sample t-test assuming equal variances.

Microsoft Excel was further utilized to visualize the difference in dynamic cross-correlation matrix (DCCM) between variant and native myocilin in order to study the effects of the E414K substitution on other residues of the OLF domain. The average values for both the variant and native proteins were calculated, and the difference between the two was found to produce a heat map. The PDB identifier of myocilin (4WXQ) was entered into the ConSurf-DB program to estimate the pathogenicity of the E414K variant by calculating the degree of variability or conservation of the glutamate at 414 across species [14]. The variant was additionally analyzed through the consensus classifier PredictSNP (developed by Jiri Damborsky and colleagues at Masaryk University, Brno, Czech Republic) by inputting the FASTA sequence of myocilin and selecting the E414K mutation to calculate the probability of the mutation being pathogenic or benign [15].

## Results

**Figure 1:**
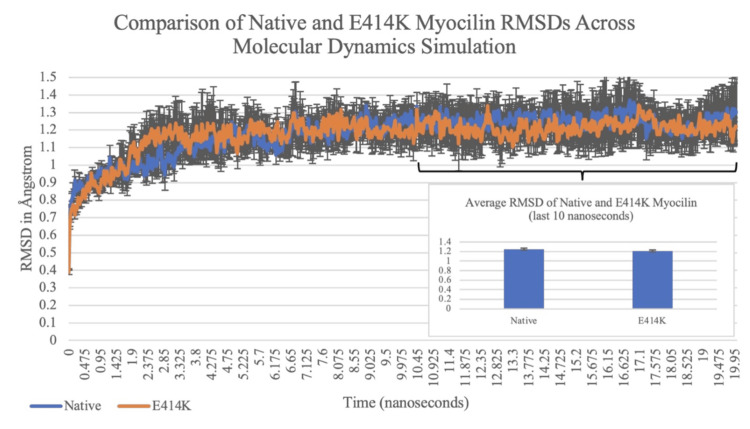
Analysis of native and E414K myocilin root-mean-square deviations (RMSD) across molecular dynamics simulation The graph compares the root-mean-square deviations (RMSD) of the native myocilin proteins (blue) and E414K myocilin proteins (orange) across the 20-nanosecond simulations. The inset column graph demonstrates the mean RMSD values of the native and E414K replicates in the last 10 seconds of the simulation (p > 0.05).

The RMSD of the native and variant myocilin proteins across the 20-nanosecond simulations was qualitatively compared on a graph to determine if the E414K mutation caused a significant structural change to myocilin’s OLF domain. Figure 1 illustrates minimal visible differences between the two graphs, especially from 10 nanoseconds to 20 nanoseconds when the protein conformations had stabilized. A two-sample t-test assuming equal variances was calculated from the RMSD averages of the variant myocilin replicates and native replicates of the last 10 seconds for a quantitative analysis of potential structural changes in the OLF domain. As shown in the inset column graph of Figure 1, the mean RMSD value of the native myocilin protein was 1.251 Angstroms (Å), and the mean RMSD value of the E414K variant was 1.215 Å. Because the two-tailed p-value (p = 0.788) was greater than the alpha-value of 0.05, the difference between the two means was not statistically significant (Figure 1).

**Figure 2:**
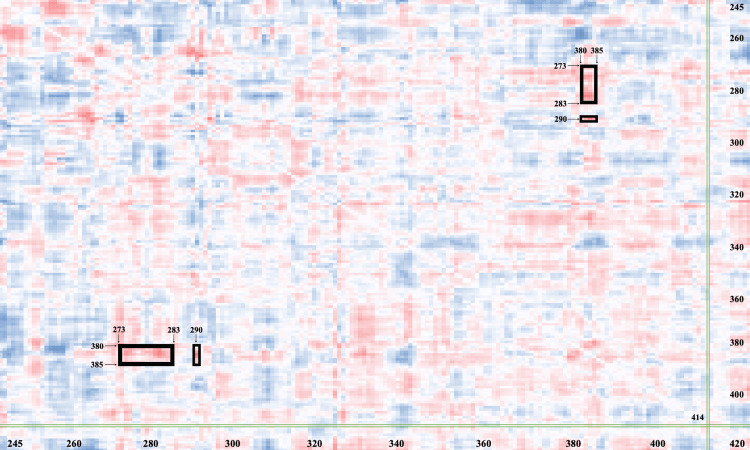
Dynamic cross-correlation matrix (DCCM) heat map of native and E414K myocilin residue interactions The heat map visualizes the correlation of 180 residues of the olfactomedin (OLF) domain and changes in residue interactions between the native and E414K myocilin proteins. The X- and Y-axes represent the residue indices of the protein sequence. The glutamate-to-lysine substitution at 414 is indicated by green vertical and horizontal boxes.  Dark red areas represent residue interactions within the OLF domain that have been significantly affected by the variant. The interactions of residues 273-283 and 290 with residues 380-385, which are significantly altered by the E414K substitution, are indicated by black boxes.

A DCCM heat map was produced to visualize the effects of the E414K substitution on other residues and the interactions between AAs in the C-terminal OLF domain. Areas of darker color represent significant changes between variant and native residue interactions in the OLF domain. Residues 273 to 283 and residue 290, highlighted with boxes in Figure 2, demonstrate significant alterations in their interactions with residues 380-385 due to the E414K substitution. The mutation may be interfering with either the internal interactions of the selected residues or the external interactions between myocilin and adjacent proteins (Figure 2).

**Figure 3:**
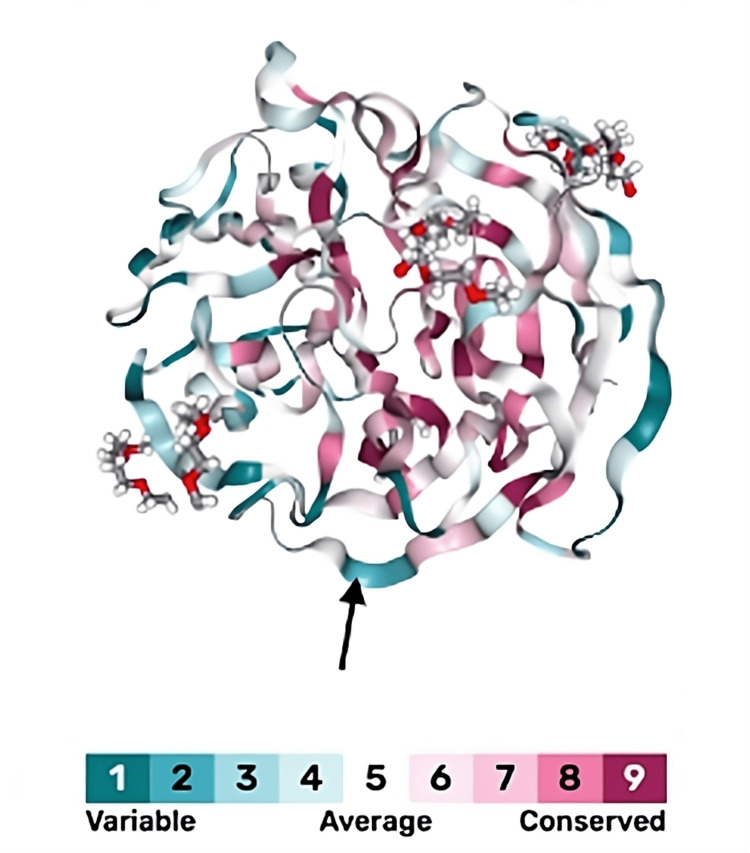
Evolutionary analysis of myocilin on ConSurf-DB The structure of myocilin, with evolutionary patterns of its residues, was generated from the ConSurf-DB program [14]. Blue colors represent residues that are variable and hence more likely to tolerate substitutions, while residues in red are conserved across species and more likely to be pathogenic. The glutamate residue at 414 (black arrow) is relatively variable, with a score of 2.

The evolutionary analysis of myocilin was visualized with the ConSurf-DB (developed by Adi Ben Chorin and colleagues at Tel Aviv University, Tel Aviv-Yafo, Israel) program [14]. As depicted on the scale, darker blue colors represent residues that are more variable across species, suggesting that these sites may be more tolerant to substitutions. In contrast, residues in red are highly conserved across species and play a critical role in protein function or structure, and mutations at these sites are thus more likely to be deleterious. The glutamate at position 414 of myocilin (black arrow) is dark blue, suggesting that the residue is variable and that mutations of the AA (i.e., E414K) will likely be neutral (Figure 3).

The E414K mutation was entered into the consensus classifier PredictSNP to calculate the pathogenicity of the variant [15]. As seen in Table 1, five out of the six predictive programs (MAPP, PolyPhen-1, PolyPhen-2, SIFT, SNAP) determined the myocilin E414K variant to be neutral or benign, with expected accuracies ranging from 61% to 77%. PhD-SNP was the only program that suggested the mutation was deleterious or pathogenic, with 77% accuracy. After combining the predictions for each tool into the PredictSNP prediction by weighted majority vote consensus, PredictSNP concluded the E414K substitution as neutral with a confidence of 75% (Table 1).

**Table 1:** Results of myocilin E414K input into PredictSNP The E414K mutation was entered into PredictSNP [15] to estimate the pathogenicity of the substitution. Five out of six predictive programs determined the myocilin E414K variant to be neutral with relatively high accuracy; PhD-SNP predicted E414K to be deleterious. The consensus classifier PredictSNP calculated the mutation as neutral with 75% accuracy.

Program	Result	Observed Accuracy
MAPP	Neutral	74%
PhD-SNP	Deleterious	77%
PolyPhen-1	Neutral	67%
PolyPhen-2	Neutral	64%
SIFT	Neutral	77%
SNAP	Neutral	61%
PredictSNP Consensus Prediction	Neutral	75%

## Discussion

In the current study, we analyzed a variant of uncertain significance of myocilin to determine its effect on the pathogenesis of POAG. As mentioned, mutations in the C-terminal OLF domain of myocilin cause aggregation of insoluble and soluble misfolded proteins within the trabecular meshwork, leading to an obstruction of aqueous humor outflow at the iridocorneal angle [3,6]. We selected to study a glutamate-to-lysine mutation at residue 414, one of the 114 variants of myocilin with unknown pathogenicity. Multiple studies were performed to investigate the myocilin E414K variant thoroughly: quantitative and qualitative analyses of the RMSDs of the native myocilin protein and variant myocilin, production of a DCCM heat map, research of the evolutionary conservation patterns of native myocilin with the ConSurf-DB program, and examination of collective predictions of the myocilin mutation through the consensus classifier PredictSNP.

The simulation results were compared via RMSD graphs of the native and variant proteins across the simulation. As demonstrated in Figure 1, both trends were nearly identical on qualitative analysis, demonstrating the absence of structural changes in the OLF domain that were anticipated by the mutation. No visible differences are recognized between the two graphs, especially from 10 nanoseconds to 20 nanoseconds, when the protein conformations had stabilized. These findings altogether represent a minimal and insignificant impact of the E414K substitution on the OLF domain structure. We also calculated the RMSD averages of the E414K myocilin replicates and the native replicates of the last 10 seconds of the simulations to produce a two-sample t-test for quantitative analysis of possible structural changes in the OLF domain, and therefore, potential contribution to the aggregation of misfolded myocilin proteins involved in POAG. The p-value was greater than the alpha value of 0.05 (p-value = 0.788), suggesting that there was not a statistically significant difference between the RMSD averages of the two proteins, and therefore, no significant difference in the native and variant structures (Figure 1) [16].

These findings initially appear to contrast with the results of the DCCM heat map, where the E414K mutation was shown to affect a few interactions between myocilin residues within the crucial OLF domain (Figure 2). The interactions of residues 273-283 and 290 with residues 380-385 were significantly affected by the mutation, potentially suggesting a deleterious nature of the E414K substitution. This change may have disrupted either internal relationships of the residues within the functional OLF domain or interactions of myocilin with other molecules or proteins. For example, mutations that affect aspartate-273 significantly decrease the thermal stability of myocilin [17]. 273 to 283 is also known to be a proline-rich region of the OLF domain (e.g., proline-274, -276) and is associated with tryptophan residues that stabilize the hydrophobic core [18]. Hence, it is likely that the E414K substitution disrupted the hydrophobic interactions or destabilized the internal packing of tryptophan with other nearby aromatic residues.

In regard to the sequence of AA 380-385, aspartate-380 is a calcium-binding ligand that is essential to the structural integrity of the OLF domain. Mutations of the aspartate at 380 are often pathogenic and associated with the development of glaucoma, as substitutions interfere with calcium binding and cause subsequent misfolding of the myocilin proteins [17]. While our study did not specifically induce mutations of aspartate-380, there lies the possibility that the E414K substitution may have indirectly disrupted the calcium-binding ability at this site [17]. Furthermore, the specific function of myocilin residues 381-385 in the literature is limited; however, we hypothesize that this region is involved in hydrophobic interactions with residues 273-283. Positions 381-383 consist of the nonpolar AAs leucine, alanine, and valine [9]. Given the observed effects of the E414K mutation on interactions between residues 273-283 and 380-385 in Figure 2, it is likely that these hydrophobic residues interact with the proline-rich region of AA 273-283 to help stabilize protein structure.

Although the E414K mutation caused significant alterations in the interactions between and within regions 273-283, 290, and 380-385, a broader examination of the DCCM heat map reveals that no other residues within the OLF domain were affected in a significant manner by the glutamate-to-lysine mutation. While these specific alterations do not appear to be as detrimental as other potential mutations to the overall integrity of the OLF domain, their cumulative effect may nonetheless have subtle impacts on myocilin stability or function that are not fully revealed in the DCCM heat map analysis. Hence, the potential for the interactions of residues 273-283, 290, and 380-385 to influence the misfolding and aggregation of myocilin proteins remains an area for further exploration.

The ConSurf-DB program was utilized to examine the evolutionary patterns of myocilin and understand the structural and functional importance of AAs within the protein, specifically the glutamate residue at position 414. According to Ben Chorin et al., residues that are highly conserved across species evolve at a slower rate compared to other positions. These residues exhibit functional importance and are generally found within regions that are essential for protein structure, ligand binding, or the function of the protein [14]. Therefore, substitutions at these positions are likely to be pathogenic and disrupt the protein’s activity or significantly alter interactions between AAs within the protein, leading to disease [19]. On the other hand, variable residues are positions that may tolerate substitutions without affecting the protein structure or activity. While not directly contributing to the protein’s function, these residues are involved in protein-protein interactions and interactions with other molecules or AAs [20]. As seen in Figure 3, the glutamate residue at 414 was variable and not conserved across species. Because of its variable nature, substitutions at this glutamate, such as the E414K mutation, are unlikely to be deleterious to myocilin’s inherent function or cause instability of the protein.

PredictSNP was used to estimate the pathogenicity of the myocilin E414K substitution by combining the results of six established prediction tools: MAPP, PhD-SNP, PolyPhen-1, PolyPhen-2, SIFT, and SNAP. Table 1 demonstrates the nearly consistent prediction of E414K to be a neutral mutation across multiple programs, with observed accuracies as high as 77%. PhD-SNP was the only tool to predict the substitution as deleterious, and it also had high accuracy [15]. This discrepancy in prediction may be explained by differences in the algorithmic frameworks of these programs. For example, PhD-SNP utilizes a support vector machine (SVM) that is trained on datasets of known disease-associated and neutral mutations, focusing on evolutionary conservation [21]. Meanwhile, tools like SIFT and PolyPhen-2 use sequence homology, protein structures, and phylogenetic information to predict the effect of a substitution on protein function [22,23]. Ultimately, the weighted majority vote consensus by PredictSNP determined the E414K substitution to be a benign mutation. These findings correlate with those of the RMSD analysis, DCCM heat map, and ConSurf-DB study, which suggests that the E414K substitution is highly unlikely to play a role in the pathogenesis of POAG. The mutation has consistently shown no significant impact on the structure or function of myocilin, and it can be confidently deduced from our results that the E414K substitution within the C-terminal OLF domain does not contribute to the misfolding and aggregation of the mutated myocilin proteins observed in POAG.

The primary limitation of our study is the scope of the analysis, as our project focused on a single variant of uncertain significance of myocilin. While any findings contribute to the broader effort to uncover the pathogenicity of all myocilin variants, our study is brief and investigates only one VUS, which provides a small impact on the overall picture. Furthermore, we did not analyze the interaction of the myocilin E414K variant with other proteins. Although the E414K substitution did not significantly affect the inherent structure or function of myocilin, there remains the possibility that the mutation may alter myocilin’s protein-protein interactions or induce misfolding in other proteins. Another limitation of our study is the small sample size, as only three replicates were created for each myocilin structure (native and variant). Smaller sample sizes reduce statistical power, and although the difference between the RMSD means of the two myocilin proteins was not statistically significant, analyzing more replicates may either reinforce this conclusion or reveal a statistical significance between the means.

To address these issues in future studies, multiple VUS should be analyzed collectively rather than one variant. For example, dozens of variants may be studied in a single project through in vitro assays and predictive programs. Not only does this technique increase the effectiveness of the study by analyzing multiple VUSs, but it also enables the investigation of potential interactions between multiple variants simultaneously. Moreover, future research could examine the interactions of myocilin variants with other proteins (e.g., neighboring myocilin structures), going as far as simulating two different myocilin proteins, each with a distinct VUS substitution, and investigating their relationships. Finally, a larger number of replicates should be analyzed to improve the statistical power and robustness of the study.

## Conclusions

Based on the findings of our study, we conclude that the glutamate-to-lysine substitution at residue 414 in myocilin is highly unlikely to play a role in the pathogenesis of POAG. Analysis across multiple programs has consistently demonstrated the benign nature of the E414K variant. While our study is brief and limited in scope, we hope that our findings will encourage further, more comprehensive studies on myocilin and its variants, ultimately contributing to enhanced diagnostics and treatments for patients with hereditary POAG.
